# Oocyte growth, follicular complex formation and extracellular-matrix remodeling in ovarian maturation of the imperial zebra pleco fish *Hypancistrus zebra*

**DOI:** 10.1038/s41598-018-32117-7

**Published:** 2018-09-13

**Authors:** Ivana Kerly S. Viana, Liziane A. B. Gonçalves, Maria Auxiliadora P. Ferreira, Yanne A. Mendes, Rossineide M. Rocha

**Affiliations:** Institute of Biological Sciences, Universidade Federal do Pará, Belém, Pará Brazil

## Abstract

This contribution describes the growth of oocytes, addressing the formation of structures that compose the follicular complex, as well as the remodeling of the extracellular matrix, specifically laminin, fibronectin and type IV collagen during gonadal maturation. Thirty-seven females of the Acari zebra fish, *Hypancistrus zebra* were captured and the ovaries were submitted to histological processing for light and electron microscopy and immunohistochemistry techniques. Oogonia and four stages (I – IV) of oocytes were distinguished, and structures such as the postovulatory follicle and atretic oocytes (initial and advanced atresia) were observed. The follicular complex consists of the mature oocyte, zona radiata (Zr1, Zr2 and Zr3), follicular cells, basement membrane and theca. During oocyte growth, proteins of the extracellular matrix showed different intensities of staining. Based on these observations, a model of oocyte growth is proposed to define specific characteristics of the oocyte and the remodeling of the extracellular matrix in the ovary of *H*. *zebra*. This model of oocyte growth can be extended to other species of ornamental fishes. This study contributes data for induced fertilization and eventual conservation of this species.

## Introduction

Oocyte growth in teleost fishes has been widely investigated in order to elucidate the various morphological changes that occur during reproduction. This reproductive process consists of the multiplication of the oogonia and the differentiation and release of mature oocytes^1–5^.

During gonadal development the oocytes gradually produce yolk globules and the cell volume increases, the nucleus moves toward the cell periphery and the micropyle is formed. The follicular cells undergo molecular changes with the extracellular matrix reorganization and the theca appears^6–8^. These modifications occur in the layers that surround the oocyte, for example the zona radiata, follicular cells, basement membrane and theca, and together with the mature oocyte form the follicular complex, FC^9^. The FC is important for reproduction and is observed in different orders, Labriformes^10^, Perciformes^11^ and Siluriformes^12^. However, information concerning the emergence of the layers that form this complex is limited for teleost fishes.

The extracellular matrix is composed of fibrillar proteins, glycoproteins, and proteoglycans that provide structural support for the cells and control the transport of nutrients, hormones and other signaling substances from the extracellular environment^13,14^. Among the extracellular-matrix components, laminin and collagen type IV are important; these are specific to the basement membrane or external layer of cells and provide cell-matrix adhesion. The glycoprotein fibronectin also provides adhesion and support for the matrix. All components are involved in growth, differentiation and cell migration, i.e., the extracellular matrix aids in maturation of the ovarian germ cells^13,15^. Although oocyte growth is important, information on this process in ornamental fishes of the family Loricariidae remains limited.

The imperial zebra pleco, *Hypancistrus zebra*^16^, commonly known as the Acari zebra or commercial code L46 is a small loricariid endemic to the Xingu River basin. *Hypancistrus zebra* is widely used in the ornamental-fish trade, although its capture and marketing are illegal since this overexploited loricariid has been added to the Brazilian list of endangered species, and since 2016 because of its vulnerability was added to the list of the Convention on International Trade in Endangered Species (CITES). Study of this species is particularly critical because its habitat is located in an area where a hydroelectric dam was constructed. Our study investigated the oocyte growth and follicular complex formation in *H*. *zebra*, through structural, ultrastructural, morphometric and immunohistochemistry analyses during oocyte maturation.

## Results

### Gonadal Anatomy

**Figure 1:**
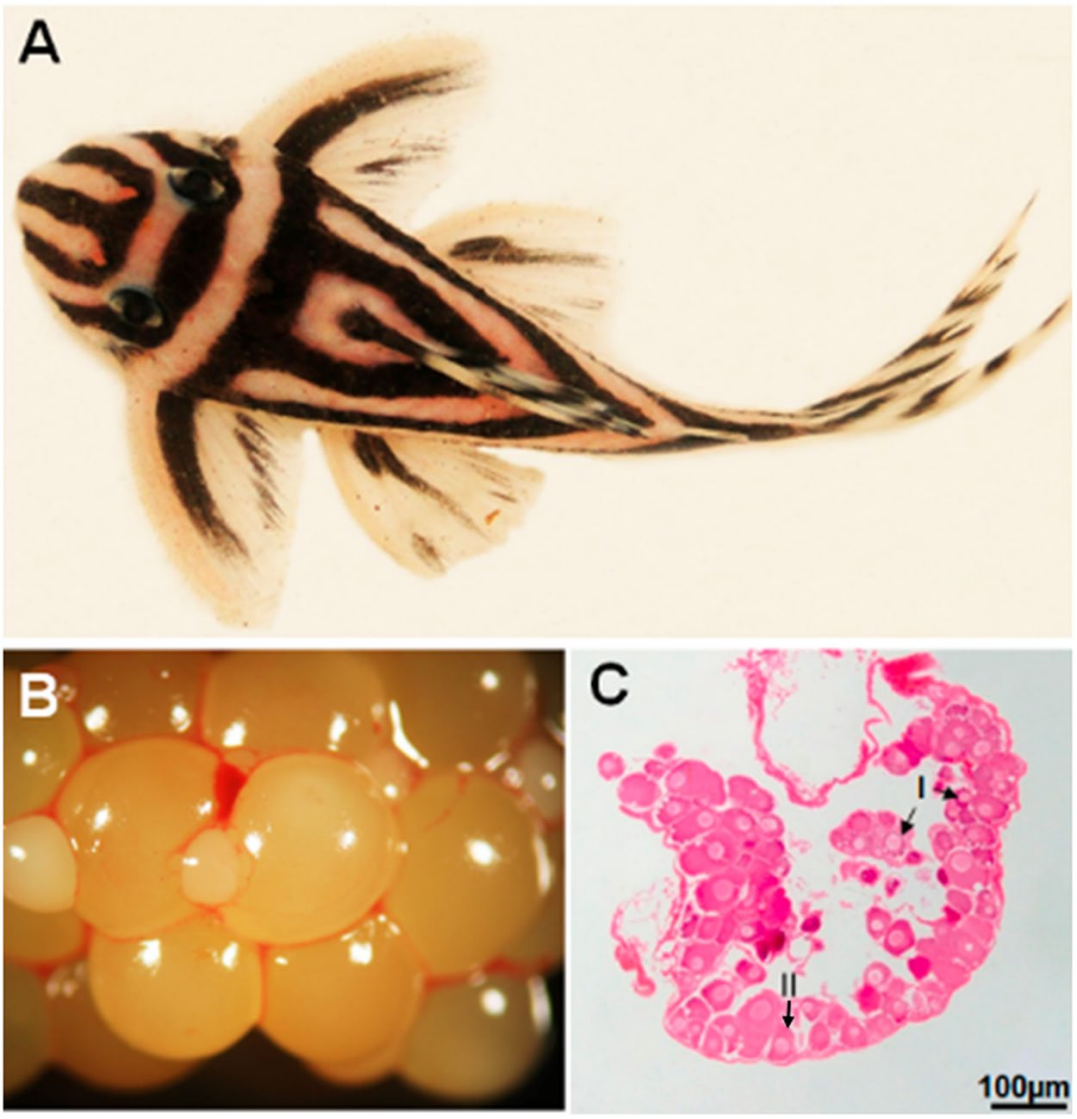
(**A**) Photograph of specimen of *Hypancistrus zebra*. (**B**) Mature ovary containing oocytes of different sizes. (**C**) ovigerous lamellae in maturing stage.

The ovary of *H*. *zebra* (Fig. 1A) consists of a pair of ovarian cyst-type sacculiform organs, located dorsally in the abdominal cavity and extending toward the tail, joining in the distal portion of the body to form a single gonoduct where the oocytes are released. The large mature ovary occupies almost the entire abdominal cavity, and is yellowish and highly vascularized (Fig. 1B).

### Characterization of oocyte types

**Figure 2:**
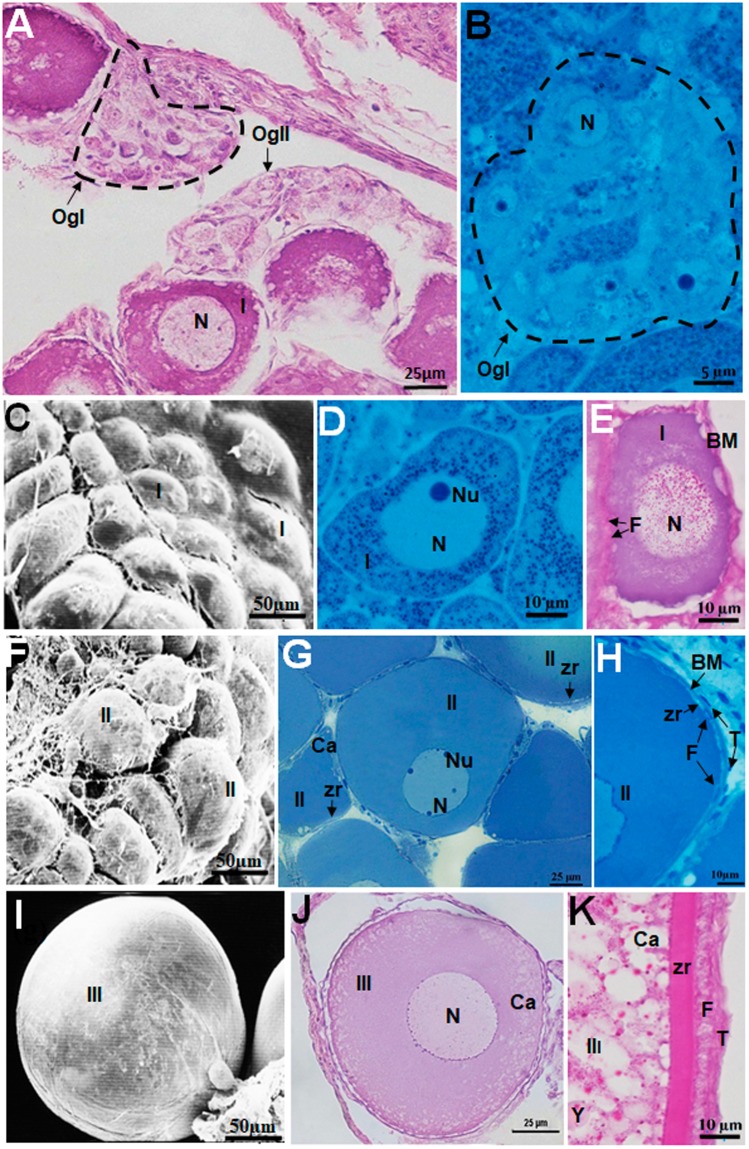
Morphology of oocyte types in *Hypancistrus zebra*. (**A**,**B**) Oogonia nest. (**C**,**F**,**I**) SEM of the surface of stage I, II and III oocytes, respectively. (**D**,**E**) Stage I oocytes with granules in the cytoplasm and nucleus. (**G**) Stage II oocyte. (**H**) Formation of the layers that surround the stage II oocyte. (**J**) Stage III oocyte. (**K**) Layers surrounding the stage III oocyte. Stains: B,D,G,H - Methylene blue. E, K - PAS/hematoxylin. A, J - HE. N: nucleus, Nu: nucleolus, OgI: Oogonia nest, OgII: Isolated oogônia, Ca: cortical alveolus, BM: basement membrane, F: follicular cell, Zr: zona radiata, Y: yolk globules, T: theca layer.

In the ovarian tissue, germ cells are surrounded by ovigerous lamellae that support the oogonia and oocytes (Fig. 1C). Oogonia may be individual (OgI) or grouped into nests (OgII) (Fig. 2A,B), and have a mean diameter of 5.97 ± 1.55 µm. Compared to the cytoplasm, oogonia have a large nucleus with only one nucleolus, which may be located in the center or at the periphery of the nucleus.

Stage I oocytes have a mean diameter of 44.07 ± 11.72 µm, the cytoplasm is homogeneous and basophilic, and the nucleus is central with a single nucleolus and is surrounded by squamous follicular cells. Granulations are visible in the cytoplasm and nucleus (Fig. 2D,E). Stage II oocytes have a mean diameter of 103.71 ± 23.32 µm and a basophilic cytoplasm that contains cortical alveoli immediately below the plasma membrane (MC); the nucleus is evident, with nucleoli scattered at the periphery of the nuclear membrane (Fig. 2G). The zona radiata (Zr) is located between the plasma membrane of the oocyte and the follicular cells (Fig. 2H). A fibrillar network surrounds stage I and II oocytes (2 C, F).

Stage III oocytes have a mean diameter of 212.20 ± 83.33 µm and a dense fibrillar network (Fig. 2I). The nucleus is central, with acidophilic cytoplasm containing yolk globules, and cortical alveoli at the periphery of the cytoplasm (Fig. 2J). These oocytes have a thick zona radiata, cuboid follicular cells and the theca (Fig. 2K).

**Figure 3:**
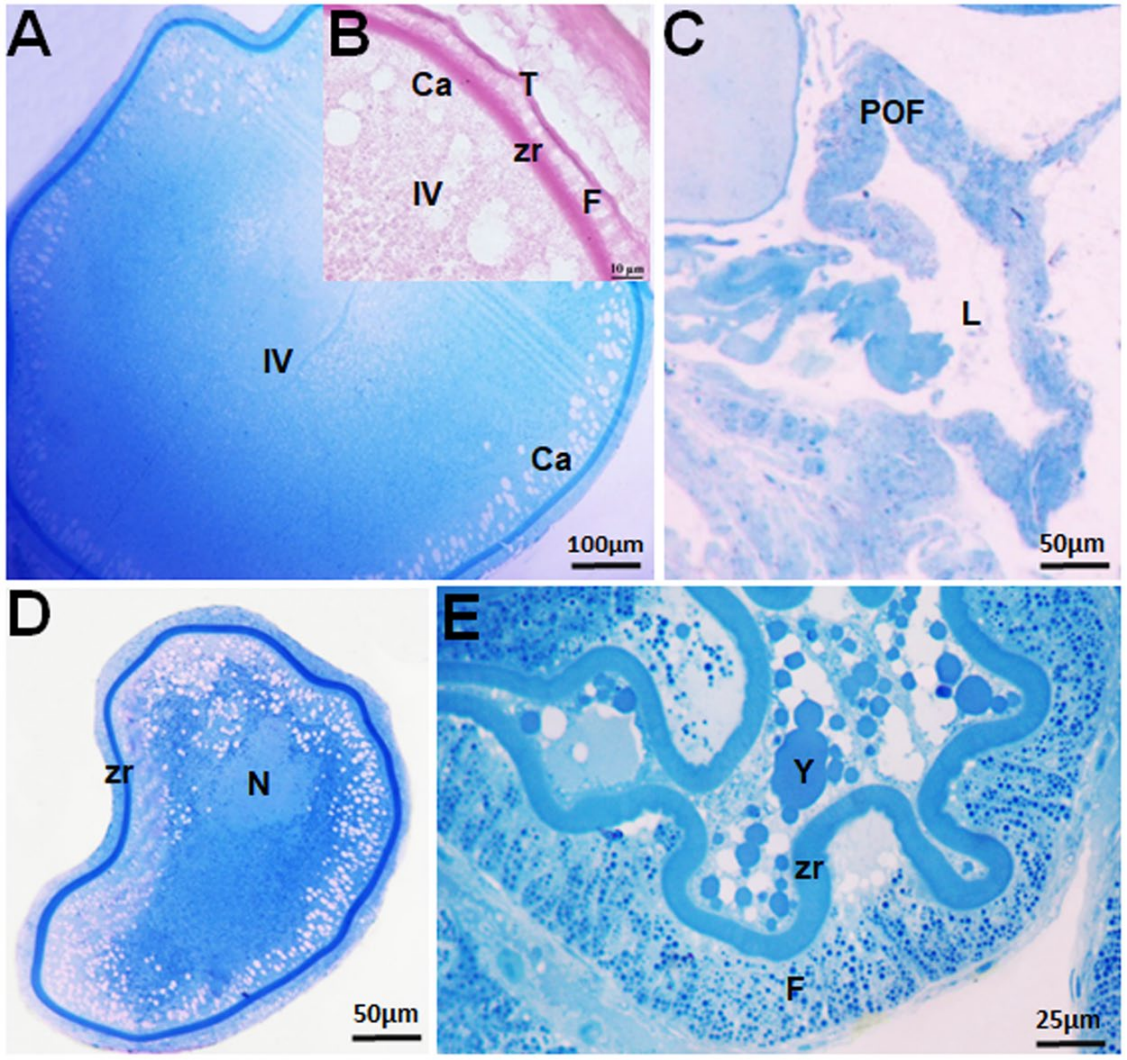
Different structures in the ovary of *Hypancistrus zebra*. (**A**) Stage IV oocyte. (**B**) The layers (Zr, F and T) that surround the stage IV oocyte. (**C**) Postovulatory follicle, showing the lumen. (**D**) Oocytes in initial atresia. (**E**) Oocyte in final atresia. Stains: A,C,D,E - Methylene blue, B - PAS/hematoxylin. N: nucleus, Ca: cortical alveolus, F: follicular cell, Zr: zona radiata, Y: yolk globules, T: theca layer, L: lumen.

**Figure 4:**
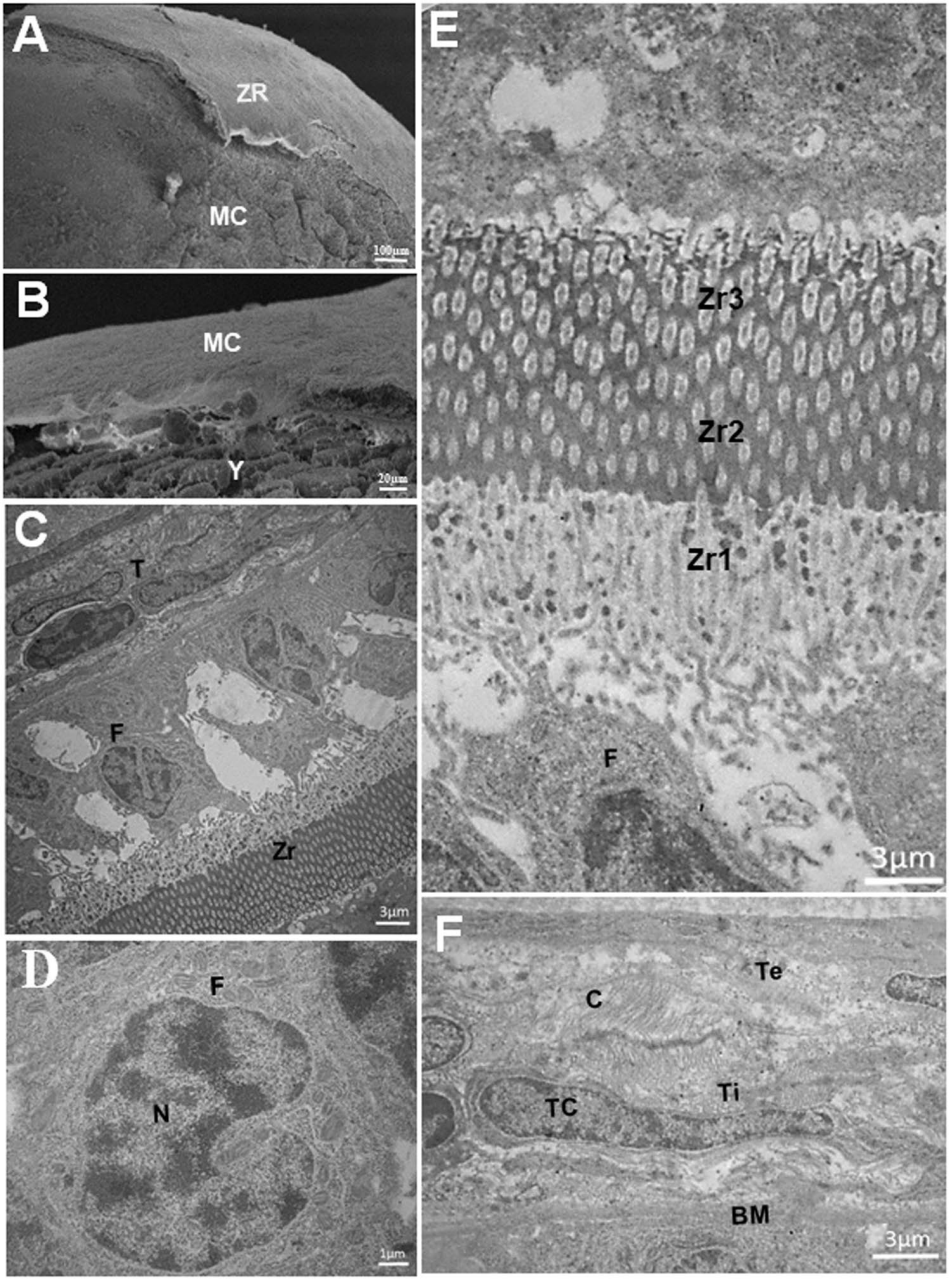
Follicular complex formation in *Hypancistrus zebra*. (**A**,**B**) SEM of surface of stage IV oocyte, showing membrane of oocyte and zona radiata (MC, Zr). (**C**) Detail of the layers that forms the follicular complex. (**D**) Follicular cell with irregular cuboid nucleus and condensed chromatin. (**E**) Subdivision of zona radiata. (**F**) Theca layer, showing internal theca with theca cell, and external theca with collagen. BM: basement membrane, F: follicular cell, Zr: zona radiata, Zr1: zona radiata 1, Zr2: zona radiata 2, Zr3: zona radiata 3, Y: yolk globules, c: collagen fibers, T: theca, Ti: internal theca, Te: external theca.

**Figure 5:**
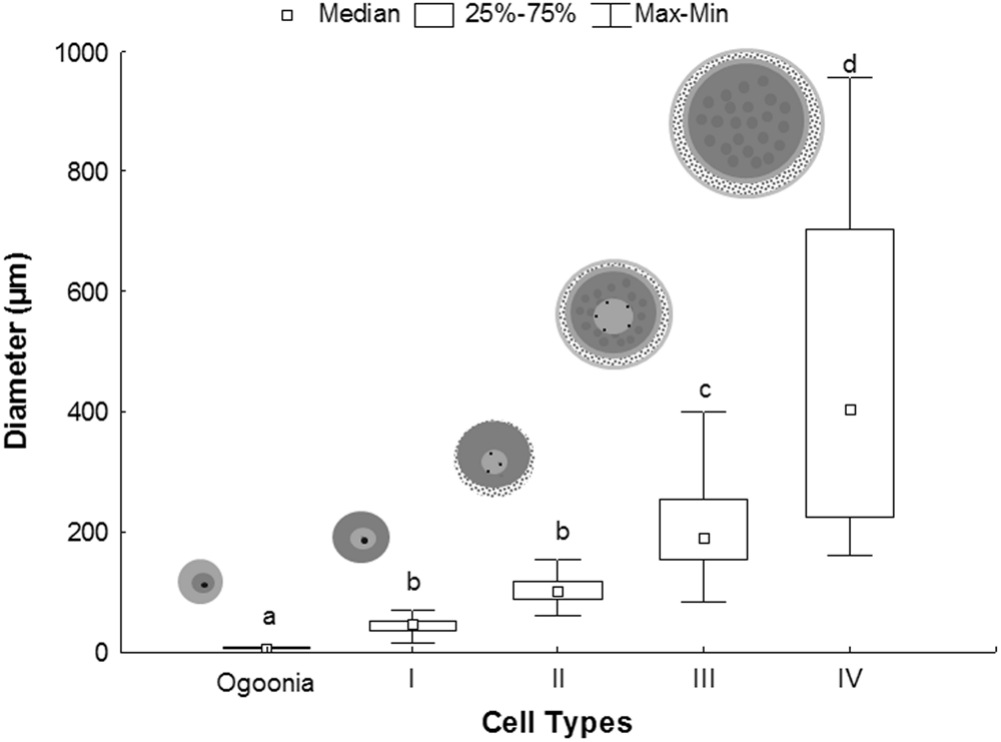
Mean diameters (µm) of cell types in *Hypancistrus zebra*.

Stage IV oocytes have a mean diameter of 487.81 ± 275.85 µm. The cytoplasm, filled with yolk globules and cortical alveoli, is located below the plasma membrane, and the nucleus migrates to the periphery of the cell (Fig. 3A,B). These cells are surrounded by the zona radiata, follicular cells and theca (Fig. 4C). The oocyte types show significant differences, except stages I and II (F = 87.26, df = 4, p < 0.0001) (Fig. 5).

The presence of postovulatory follicles (POFs) indicates that spawning has occurred. They are formed of follicular cells, basement membrane and theca. POFs are irregular in form, with a lumen, and remain in the ovary until subsequent reabsorption (Fig. 3C). The atresia was classified as initial and advanced. Initial atresia is characterized by the irregular form of the oocyte and the fragmentation of the nucleus (Fig. 3D). In advanced atresia, the nucleus is not observed, the follicular cells and theca are hypertrophied and the oocyte is totally deformed (Fig. 3E).

### Structure of the follicular complex

The formation of the follicular complex begins with the stage I oocyte and is characterized by a thin layer of squamous follicular cells. Stage II oocytes have a zona radiata, follicular cells, a basement membrane, and fusiform cells organizing outside the lamina, characterizing the emergence of the theca (Fig. 2H). Stage III and IV oocytes have the zona radiata, follicular cells, theca and basement membrane, which together form the follicular complex. These structures are thicker in stage IV oocytes (Fig. 3B).

Ultrastructurally, stage IV oocytes contain larger numbers of yolk globules in the cytoplasm (Fig. 4B). The zona radiata is subdivided into the zona radiata 1 (Zr1), zona radiata 2 (Zr2) and zona radiata 3 (Zr3). The Zr1 is in contact with the follicular cell layer through membrane projections, and is less electron-dense than the other layers. The intermediate Zr2 layer has smaller, rounded pore canals. The Zr3 layer is in contact with the oocyte membrane (MC), with larger and irregularly shaped pore canals. Zr2 and Zr3 have microvilli inside the pore canals (Fig. 4A,C,E). There is also a layer of cuboid follicular cells that have a cytoplasm filled with mitochondria and an irregularly shaped nucleus with condensed chromatin (Fig. 4D). The theca layer is subdivided into a theca interna composed of pavement cells and a theca externa composed of collagen fibers (c) (Fig. 4F).

### Immunohistochemical analysis

**Figure 6:**
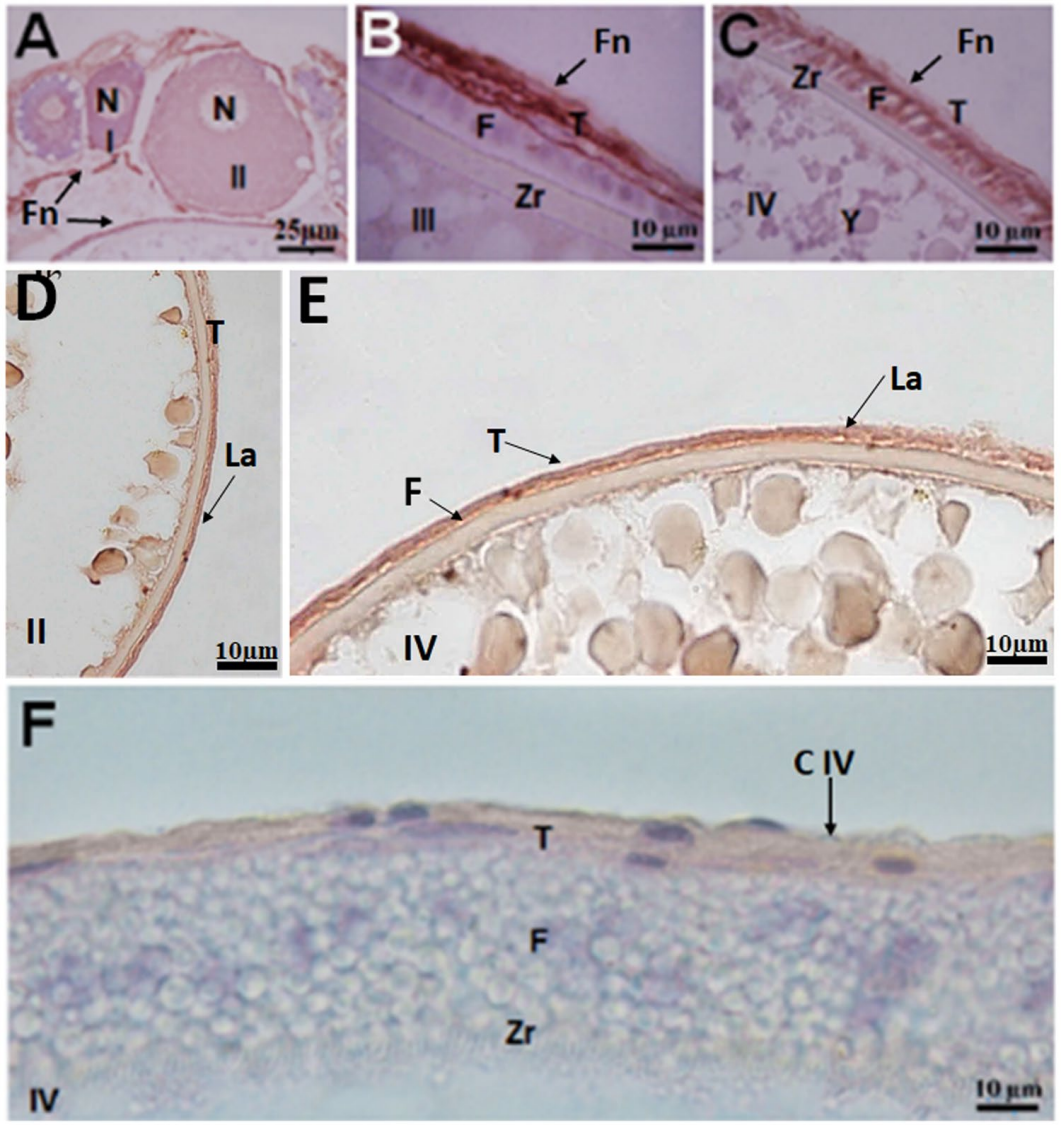
Immunohistochemistry reaction in oocytes of *Hypancistrus zebra*. (**A**–**C**) Immunolocalization of fibronectin (Fn), demonstrating reaction in basement membrane, follicular cells and theca. (**D**,**E**) Immunostaining of laminin (La) in basement membrane and theca. (**F**) Immunolocalization of collagen type IV(C IV) in theca. I: stage I oocyte, II: stage II oocyte, III: stage III oocyte, IV: stage IV oocyte, N: nucleus, BM: basement membrane, F: follicular cell, Zr: zona radiata, T: theca layer.

Immunolabeling for the extracellular-matrix protein was seen in different components of the follicular complex. Fibronectin was observed mainly in the basement membrane of types I and II oocytes (Fig. 6A), the theca layer of stage III oocytes (Fig. 6B) and the follicular cells and theca of stage IV oocytes (Fig. 6C). Laminin was observed in the follicular cells, basement membrane and theca (interna and externa), beginning with stage II oocytes and observed in the other oocyte stages (Fig. 6D,E). Collagen type IV was observed in the theca and was more evident in stage IV oocytes (Fig. 6F).

## Discussion

We characterized the structure, ultrastructure, morphometry and follicular complex formation, as well as the expression and organization of the extracellular matrix in *H*. *zebra*. The ovary of loricariids is generally large and yellowish as found in *H*. *zebra*. In this species the oocytes are large but relatively few, as in other loricariids, e.g. *Rhinelepis aspera*^17^, *Neoplecostomus microps*^18^ and *Harttia torrenticola*^19^. Generally, the presence of larger but fewer oocytes is related to the mode of reproduction and parental care of the species, which suggests a higher survival rate of the brood^19,20^.

Oogonia of *H*. *zebra* were found individually or in nests. This organization pattern is the same as in *Laetacara araguaiae*^10^, *Pseudotocinclus tietensis*^12^ and *Astyanax altiparanae*^21^. This arrangement in nests occurs through projections of follicular cells that separate the nests of other oogonia scattered through the germinal epithelium^22^. Later, the oogonia enter meiosis, initiating oocyte development. A similar appearance was found in *Oryzias latipes*, where the germ cells, oogonia and oocytes are organized in a germinal cradle^23,24^.

The early stages of oocytes of *H*. *zebra* were characterized by the presence of granulations in the cytoplasm and nucleus (Stage I) and the emergence of the zona radiata (Stage II). Ribonucleic-acid and protein synthesis start in the early stage of the cell^25–28^. Yolk globules and cortical alveoli appear and increase in stage III and IV oocytes. Five species of ostariophysan fishes show a similar sequence^19^, suggesting that these morphological characteristics are conserved in teleost species.

In the gonadal development of *H*. *zebra*, the POF had an irregular shape and central lumen. Other studies have reported the existence of a post-ovulation complex (POC), which forms immediately after ovulation, where some structures of the follicular complex such as the follicular cells, basement membrane and theca are clearly defined^11,29^. However, in *H*. *zebra* no distinction between the layers was observed. In addition to the POF, we observed follicular atresia in which we were able to distinguish initial and advanced stages; in contrast, in *Pimelodus maculatus* three stages, initial, intermediate and final atresia can be distinguished^30^. Our study established the existence of the initial and advanced atresia stages. This classification is based on the fragmentation of the nucleus and the hypertrophy of the follicular cells and theca, which occur at the same time in the follicle.

The oocytes of *H*. *zebra* vary in size (diameter 44.07–487.81 µm). The increase in the diameter of the oocytes is similar to that found in Siluriformes, including *Loricariichthys platymetopon*, *Loricariichthys* sp., *Loricaria* sp., *Hypostomus ternetzi*, *Megalancistrus aculeatus*^31^ and *Hypostomus francisci*^32^. This size variation may be related to the reproductive strategy: numerous small eggs are common in migratory species, whereas non-migratory species tend to have fewer but large eggs. Investigators distinguish between migratory species that produce more eggs to attempt to ensure the perpetuation of the species, and non-migratory species that usually have parental care and invest in the size of the eggs rather than in their quantity^20,33^. *H*. *zebra* is a non-migratory ornamental fish; hence we believe that this type of oocyte pattern suggests that the animal invests in oocyte quality with increased yolk production, ensuring nutritional reserves for the larval stage.

The emergence of FC in *H*. *zebra* begins in the stage I oocyte with the presence of the basal membrane and follicular cells, similarly to the sequence in *Sciaenops ocellatus*^9^. The subdivision of the Zr (Zr1, Zr2 and Zr3) agrees with the findings of some studies^9,34^ but differs from another^19^ that reported only two layers for the zona radiata. In *H*. *zebra* the subdivision of the zona radiata is related to the variation in the size of the pore canals (Zr1 and Zr2) and the presence of microvilli (Zr3). Some investigators have established that this layer functions to transport substances and provide resistance to abrasion^10,31^. *H*. *zebra* is a sedentary species that lives in stream rapids, suggesting that the thick zona radiata and its chemical components favor adhesion of the eggs to different substrates.

The follicular cells are squamous to cuboidal and have an irregular nucleus. Follicular cells have been found in other teleosts: cylindrical in *Trachelyopterus galeatus*, *Lophiosilurus alexandri* and *Rhinelepis áspera*^17^, cuboid in *Iheringichthys labrosus*^35^ and cuboid and prismatic in *Loricariichthys spixiiz*^36^. The morphological variation of the follicular cells depends on the maturation phase of the oocyte, since the follicular cells are one of the first elements to organize in the germinal epithelium, which is directly related to the growth and maintenance of oocytes during the reproductive cycle^26,37^.

The theca is derived from ovary cells; initially it is a thin layer, but as the oocyte grows it is possible to distinguish the internal and external theca^11^. In *H*. *zebra* the theca appears in stage II oocytes and reaches its maximum development in stage IV oocytes. This structure is associated with the follicular cells, and together they are responsible for the production of hormones that promote the maturation of the oocyte^38^. In *H*. *zebra* the theca layer also contains laminin, collagen type IV and fibronectin, suggesting that these elements have a strong influence on the organization of this layer beginning with the emergence of the follicular complex.

During gonadal maturation in *H*. *zebra*, a fibrillar network was observed around the oocyte. We believe that this arrangement supports the cells of the ovarian parenchyma during oocyte development. Some authors have reported that matrix elements are commonly associated with the growth of ovarian cells and transmit signals regulating adhesion, migration, proliferation, apoptosis, survival and differentiation^13,39–41^.

**Figure 7:**
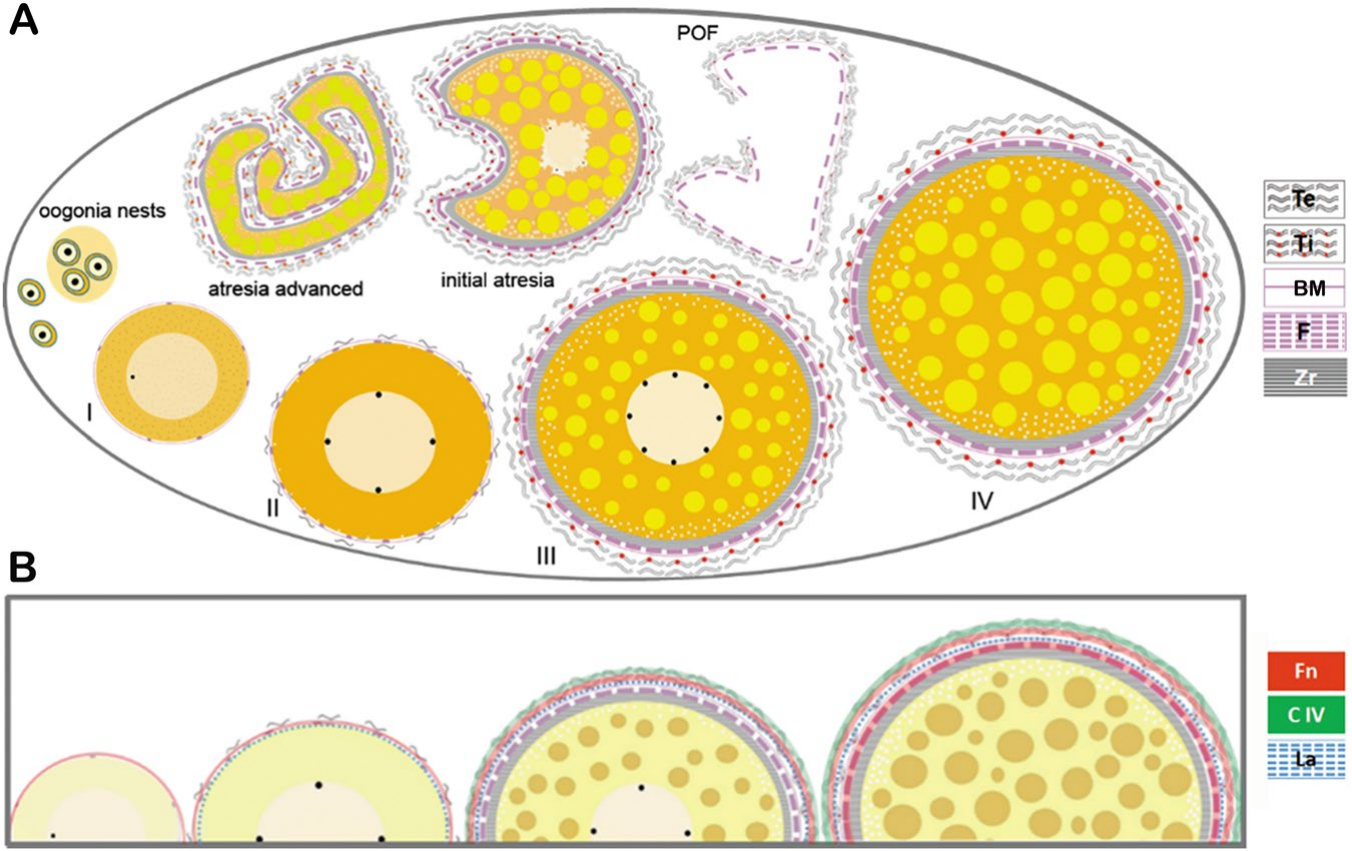
Proposed oocyte growth model and formation of follicular complex in *Hypancistrus zebra*. (**A**) Oogonia that were isolated in the ovary now form nests through projections of follicular cells, and subsequently differentiate into stage I oocytes with a peripheral or central nucleus with only one nucleolus, a basement membrane and pavement follicular cells. In stage II oocytes the zona radiata appears, as well as larger numbers of pavement follicular cells, a basement membrane and the beginning of theca organization. In stage III oocytes the nucleus contains several peripheral nucleoli and the follicle complex is composed of the zona radiata, cuboid follicular cells, theca and the mature oocyte. In stage IV oocytes the cytoplasm is filled with yolk globules, the follicle complex is thick, the internal theca formed by pavement cells is observed externally, and the external theca is composed of collagen fibers. After spawning, irregular POFs are formed by the follicular cells and theca. Oocytes at initial atresia are found in the ovary, which shows a slight loss of its rounded configuration, irregular appearance and fragmentation of the nucleus; and advanced atresia with the absence of a nucleus and a totally disorganized oocyte. (**B**) Fibronectin is found in the basement membrane of stages I and II oocytes, in the theca of stage III oocytes, and in the follicular cells and theca of stage IV oocytes. Laminin was found in the follicular cells, basement membrane and theca from stage II oocytes, and type IV collagen was found in the theca layer from stage III oocytes, accentuated in stage IV.

Based on these morphological and immunohistochemical analyses, we present a model of formation of the follicular complex in *H*. *zebra* (Fig. 7), highlighting the extracellular matrix components (laminin, fibronectin and collagen type IV).

## Materials and Methods

### Study area and preparation of specimens

A total of 37 females of *H*. *zebra* were caught in bimonthly collections in 2012 and 2013 from the Xingu River in northern Brazil (03°12′52″S, 52°11′23″W). The specimens were transported to the laboratory, anesthetized with benzocaine hydrochloride (0.1 g.L^–1^) and euthanized with sodium pentobarbital solution (60–100 mg/Kg). The gonads were removed through a ventral incision and classified into three stages: maturing, mature and spawned. The classification of oocyte development was adapted from the literature^42^. All animal experiments were approved by the National Council for Control of Animal Experimentation (CONCEA) and were performed in accordance with approved guidelines.

### Light microscopy and immunohistochemistry

Fragments of *H*. *zebra* ovary were fixed in Bouin’s solution for 24 h. The samples were then dehydrated in increasing concentrations of ethanol, cleared in xylene and infiltrated and embedded in paraffin^43^. Sections 5 μm thick were cut and stained with hematoxylin and eosin (HE) solution and periodic acid-Schiff (PAS). For immunohistochemistry, replicates of previously identified slides were deparaffinized in xylene, washed in phosphate-buffered saline (PBS) and immersed in sodium-citrate buffer heated for 25 min. Subsequently, the slides were incubated in 3% hydrogen peroxide in methanol for 30 min, blocked with 10% normal goat serum for 1 h, incubated in anti-rabbit fibronectin polyclonal primary antibody (1:200), anti-rabbit laminin (1:60) and anti-rabbit collagen type IV (1:100) for 12 h, and post-incubated in anti-rabbit IgG secondary antibody conjugated with peroxidase for 2 h. The samples were developed in DAB (3,3′diaminobenzidine) for 5 min, washed in distilled water, counterstained with hematoxylin and examined under a Carl Zeiss optical microscope (AxioStar Plus 1169151).

### Morphometry of oocytes

Samples from 100 oogonia and 100 oocytes of each type (I, II, III and IV) were analyzed. Only cells that contained a nucleus were measured. Serial sections were cut and the slides were evaluated under a photomicroscope with the software NIS-elements BR (4.00.07-bit) and measurements were made at 40X magnification. Each cell was overlaid with two dashed lines crossing at right angles in the middle of the cell, and the length of the segment of the line over that diameter of the cell was measured. The mean length of the two measurements was taken as the approximate diameter of the cell (oocyte and oogonia). This method of measuring the cell diameter was adapted from the literature^42^. In our analysis, we measured the cells in two dimensions (2d), as in several previous studies^44–46^. The data were tested through one-way analysis of variance (ANOVA), followed by *a posteriori* Tukey test with 5% significance level (α).

### Transmission Electron Microscopy (TEM) and Scanning Electron Microscopy (SEM)

Fragments of ovaries were fixed in Karnovsky’s solution (4% paraformaldehyde, 2% glutaraldehyde in 0.1 M sodium cacodylate buffer, pH 7.4) for 24 h. After fixation, the fragments were washed in 0.1 M sodium cacodylate buffer, pH 7.4 and post-fixed in 1% osmium tetroxide in 0.1 M sodium cacodylate buffer, pH 7.4 for 2 h. For TEM analysis, the fragments were dehydrated in an ascending acetone series and embedded in Epon 812. Semi-thin 1 μm-thick sections were cut in a microtome, stained with 1% methylene blue and then analyzed under a Zeiss optical microscope (AxioStar Plus 1169151). Ultra-thin sections were contrasted with uranyl acetate and lead citrate and examined in a JEOL (JEM -100CX II) electron microscope. For SEM analysis, the specimens were dehydrated in a graded ethanol series (30% to 100%) and critical-point dried using CO_2_. Specimens were mounted on stubs, coated with gold and examined using a LEO 1430 SEM.

## References

[1] Murua H, Motos L (2006). Reproductive strategy and spawning activity of the European hake Merluccius merllucius (L.) in the Bay of Biscay. J Fish Biol.

[2] García-Seoane E, Bernal A, Saborido-Rey F (2014). Reproductive ecology of the glacier lanternfish Benthosema glaciale. Hydrobiologia.

[3] Costa EFS, Dias JF, Murua H (2015). Reproductive strategy and fecundity of the keystone species Paralonchurus brasiliensis (Teleostei, Sciaenidae): an image processing techniques application. Environ Biol Fishes.

[4] Gomes ID, Araújo FG, Nascimento AA, Sales A (2015). Equilibrium reproductive strategy of the armored catfish Hypostomus auroguttatus (Siluriformes, Loricariidae) in a tropical river in Southeastern Brazil. Environ Biol Fishes.

[5] Uribe MC, Grier HJ, García-Alarcón A, Parenti LR (2016). Oogenesis: From Oogonia to Ovulation in the Flagfish, Jordanella floridae Goode and Bean, 1879 (Teleostei: Cyprinodontidae). J Morphol.

[6] Quagio-Grassiotto I, Wildnera DD, Guimarães-Bassolic ACD (2014). A cytochemical approach to describe oocyte development in thefreshwater ostariophysan, Serrasalmus maculatus (Characiformes). Micron.

[7] Murata K, Conte FS, McInnis E, Fong TH, Cherr GN (2014). Identification of the Origin and Localization of Chorion (Egg Envelope) Proteins in an Ancient Fish, the White Sturgeon, Acipenser transmontanus. Biol Reprod.

[8] Abreu, M. R., Garcia, P. & Zaniboni-Filho, E. Histological characterization of oocyte developmental stages of suruvi Steindachneridion scriptum kept in captivity. *Acta Sci Anim Sci* Maringá: 351–356 (2015).

[9] Grier HJ (2012). Development of the Follicle Complex and Oocyte Staging in Red Drum, *Sciaenops ocellatus* Linnaeus, 1776 (Perciformes, Sciaenidae). J. Morphol..

[10] Santos-Silva AP, Siqueira-Silva DH, Ninhaus-Silveira A, Veríssimo-Silveira R (2015). Oogenesis in Laetacara araguaiae (Ottoni and Costa, 2009) (Labriformes: Cichlidae). Zygote.

[11] Grier HJ, Neidig CL, Quagio‐Grassiotto I (2017). Development and fate of the postovulatory follicle complex, postovulatory follicle, and observations on folliculogenesis and oocyte atresia in ovulated common snook, Centropomus undecimalis (Bloch, 1792). J. Morphol..

[12] Rodrigues-Filho JA (2017). Reproductive biology of Pseudotocinclus tietensis (Siluriformes: Loricariidae: Hypoptopomatinae), a threatened fish species. International. Journal of Aquatic Biology..

[13] Rodgers RJ, Irving-Rodgers HF, Russell DL (2003). Extracellular matrix of the developing ovarian follicle. Reproduction.

[14] Kim SH, Turnbull J, Guimond S (2011). Extracellular matrix and cell signalling: the dynamic cooperation of integrin, proteoglycan and growth factor receptor. J Endocrinol.

[15] Beck K, Hunter I, Engel J (1990). Structure and function of laminin: anatomy of a multidomain protein. FASEB J.

[16] Isbrucker IJH, Nijssen H (1991). Hypancistrus zebra, a new genus and species of uniquely pigmented ancistrine loricariid fish from the Rio Xingu, Brazil (Pisces: Siluriformes: Loricariidae). Ichthyol Explor Fres.

[17] Melo RMC (2011). Comparative Morphology of the Gonadal Structure Related to Reproductive Strategies in Six Species of Neotropical Catfishes (Teleostei: Siluriformes). J Morphol.

[18] Brito MFG, Lazzarotto H, Caramaschi EP (2016). Life-history features of a rapids-dwelling loricariid catfish from Atlantic forest streams, Brazil. Biota Neotrop.

[19] Melo RMC (2017). Comparative morphology of the reproductive system of seven species of ostariophysan fishes from the upper Das Velhas River, Brazil. Journal of Morphology..

[20] Winemiller KO, Rose KA (1992). Patterns of life-history diversification in north american fishes: implications for population regulation. Can J Fish Aquat Sci.

[21] Cassel M (2017). Ovarian development and the reproductive profile of Astyanax altiparanae (Teleostei, Characidae) over one year: Applications in fish farming. Theriogenology..

[22] Quagio-Grassiotto I, Grier H, Mazzoni TS, Nobrega RH, Amorim JPA (2011). Activity of the ovarian germinal epithelium in the freshwater catfish, Pimelodus maculatus (Teleostei: Ostariophysi: Siluriformes): germline cysts, follicle formation and oocyte development. J Morphol.

[23] Nakamura S (2010). Identification of germline stem cells in the ovary of the teleost medaka. Science.

[24] Nishimura T (2018). Germ cells in the teleost fish medaka have an inherent feminizing effect. PLoS genetics.

[25] Selman K, Wallace RA (1989). Cellular aspects of oocyte growth in teleosts. Zool Sci.

[26] Grier H (2000). Ovarian germinal epithelium and folliculogenesis in the Common Snook, Centropomus undecimalis (Teleostei: Centropomidae). J Morphol.

[27] Patiño R, Sullivan CV (2002). Ovarian follicle growth, maturation, and ovulation in teleost fish. Fish Physiol Biochem.

[28] Jalabert B (2005). Particularities of reproduction and oogenesis in teleost fish compared to mammals. Reprod Nutr Dev.

[29] Cassel M, Camargo MP, Jesus LWO, Borella MI (2017). Involution processes of follicular atresia and post-ovulatory complex in a characid fish ovary: a study of apoptosis and autophagy pathways. Journal of Molecular Histology..

[30] Paschoalini AL (2013). Reproduction of Pimelodus maculatus (Siluriformes: Pimelodidae) in three section of Grande River basin, downstream Porto Colombia dam, south-eastern Brazil. Neotrop Ichthyol.

[31] Susuki HI, Agostinho AA, Winemiller KO (2000). Relationship between oocyte morphology and reproductive strategy in loricariid catfishes of the Paraná River, Brazil. J Fish Biol.

[32] Sales CF (2016). Biological variables of Hypostomus francisci (Siluriformes: Loricariidae) from Itapecerica River, Minas Gerais State, Brazil. An Braz Acad Sci.

[33] Kolm N, Ahnesjo I (2005). Do egg size and parental care coevolve in fishes?. J Fish Biol.

[34] Jiang YQ, Zhang TT, Yang WX (2010). Formation of zona radiata and ultrastructural analysis of egg envelope during oogenesis of Chinese perch Siniperca chuatsi. Micron.

[35] Santos JE (2006). Ovarian follicle growth in the catfish Iheringichthys labrosus (Siluriformes: Pimelodidae). Tissue and Cell.

[36] Duarte S, Araújo FG, Sales A, Bazzoli N (2007). Morphology of Gonads, Maturity and Spawning Season of Loricariichthys spixii (Siluriformes, Loricariidae) in a Subtropical Reservoir. Braz Arch Biol Technol.

[37] Quagio-Grassiotto I, Guimarães ACD (2006). Follicular ephitellium, theca and egg envelope formation in Serrasalmus spilopleura (Teleost, Characiformes, Characidae). Acta Zool.

[38] Senthilkumaran B, Yoshikuni M, Nagahama Y (2004). A shift in steroidogenesis occuring in ovarian follicles prior to oocyte maturation. Mol Cell Endocrinol.

[39] Berkholtz CB, Lai BE, Woodruff TK, Shea LD (2006). Distribution of extracellular matrix proteins type I collagen, type IV collagen, fibronectin, and laminin in mouse Folliculogenesis. Histochem Cell Biol.

[40] Thomé R, Santos HB, Sato Y, Rizzo E, Bazzoli N (2010). Distribution of lamininb2, collagen type IV, fibronectina and MMP-9 in ovaries of the teleost fish. J Mol Histol.

[41] Bonnans C, Chou J, Werb Z (2014). Remodelling the extracellular matrix in development and disease. Nature reviews Molecular cell biology..

[42] Elkouby YM, Mullins MC (2017). Methods for the analysis of early oogenesis in Zebrafish. Developmental biology..

[43] Prophet, E. B., B. Mills, J. B. Arrington & L. H. Sabin. Métodos Histotecnológicos (eds Prophet, E. B. *et al*.) Ch 5, 1–280 (El Registro de Patologia de los Estados Unidos de America, 1995).

[44] Maria AN, Orfão LH, Rizzo E, Ninhaus-Silveira A, Viveiros AT (2014). Histochemical and morphological features of biopsied and stripped oocytes from the Brazilian endangered teleost pirapitinga, Brycon nattereri (Characiformes). Zygote.

[45] Faustino F, Makino LC, Neumann E, Nakaghi LSO (2015). Morphological and morphometric aspects of early life stages of piabanha Brycon gouldingi (Characidae). Journal of fish biology.

[46] Ismail RF, Mourad MM, Farrag MM (2018). Gonadal development and hermaphroditism of bluespotted seabream, Pagrus caeruleostictus (Valenciennes, 1830) from the Mediterranean Sea, Egypt. The Egyptian Journal of Aquatic Research.

